# Chemical Composition and Preliminary Antimicrobial Activity of the Hydroxylated Sesquiterpenes in the Essential Oil from Piper barbatum Kunth Leaves

**DOI:** 10.3390/plants9020211

**Published:** 2020-02-06

**Authors:** Paco Noriega, José Ballesteros, Alejandra De la Cruz, Tatiana Veloz

**Affiliations:** 1 Group of Research and Development in Science Applied to Biological Resourses, Universidad Politécnica Salesiana, Avenida 12 de Octubre N2422 y Wilson, Quito 170109, Ecuador; mde@ups.edu.ec (A.D.l.C.); jvelozj@est.ups.edu.ec (T.V.); 2 Biotechnology Engineering, Universidad Politécnica Salesiana, Km 19.5 Via a la Costa, Guayaquil 090901, Ecuador; jballesterosl@ups.edu.ec

**Keywords:** *Piper barbatum* Kunth, antimicrobial compounds, essential oil, β–eudesmol, 10-epi-γ-eudesmol

## Abstract

This study evaluates the antimicrobial and antifungal potential of the essential oil extracted from a species located in the Andes of Ecuador, *Piper barbatum* Kunth, known as “cordoncillo” or “allupa”, used by the Quichua people as an antibacterial plant for washing female genitalia in cases of infection. The most abundant molecules in the essential oil were: α- phellandrene (43.16%), limonene (7.04%); some oxygenated sesquiterpenes such as: trans-sesquisabinene hydrate (8.23%), elemol (7.21%) and others. The evaluation of antimicrobial activity showed activity in all the strains analyzed; however, those in which MIC values are considered to be very strong (less than 500 µg/mL) are: *Staphylococcus aureus* 264 µg/mL, *Streptococcus mutans* 132 µg/mL, *Candida albicans* 132 µg/mL and *Candida tropicalis* 264 µg/mL. Antimicrobial bioautography defines which molecules are responsible for the activity; thus, it was possible to establish the chromatographic regions of = 0.02 and Rf = 0.04, as those with active molecules. It was established that 4 hydroxylated sesquiterpene molecules are involved: elemol (7.21%), trans-sesquisabinene hydrate (8.23%), β–eudesmol (3.49%) and 10-epi-γ-eudesmol (1.07%); the last two being the most active. The aim of this manuscript is to analyze both the ancestral knowledge of the Quichua people of Ecuador, and the chemical-biodiversity of the Andean forest ecosystem, in order to provide new raw materials of pharmaceutical interest.

## 1. Introduction

The Andes mountain range crosses Ecuador from north to south and creates an ecosystem of high biodiversity, the reason for which many Andean species are interesting from the medical point of view [1,2,3].

The Piperaceae family is typical of tropical America, where there are about 700 species, out of which 441 are found in Ecuador, with 134 endemic species [4]. *Piper barbatum* Kunth is a native shrub of the Andes, and it is very common in the montane forests, and it lives with other species typical of this ecosystem [5]. The Spanish name of the plant is “cordoncillo” and the Quichua name is “allupa” [6], and it is traditionally used for: headache, stomach pain, vaginal infections, dermatitis, disinfectant and plant to clean the body of bad vibes [6,7].

Chemical and pharmacological studies in the species are scarce. A study conducted in its essential oil highlights the presence of: 2-methylene-4,5 dioxy-propiophenone and methoxy-4,5-methylenedioxy-pmpiophenone isomer II [8]; another study assesses its anticancer potential [9].

Several essential Piperaceae oils from Ecuador have been shown to have high antimicrobial and antifungal activity, as an example, there are species such as: *Piper pubinervulum* [4], *Piper aduncum* and *Piper obliquun* [10], *Piper carpunya* [11] and *Piper lenticellosum* [12].

Since one of the most widespread ancestral uses of the plant is related to its antimicrobial qualities, this manuscript seeks to evaluate this capacity in its essential oil, and also to identify molecules responsible for it by using bioautographic techniques, which have proven to be useful in these assessments [13,14]

It is very common for antibiotics to be less and more effective in the fight against infections and diseases related to bacteria and pathogenic fungi [15]; hence, turning the look to natural products could be of great use in improving the health of people.

## 2. Results

### 2.1. Obtaining of the Essential Oil

High amounts of essential oil was obtained in *P. barbatum* fresh leaves were used, the average yield was 0.902 % ± 0.032, and usually, the yield of essential oil in a plant is between 0.1 to 1% by dry weight [16]. In the oil, it had a density of 1.013 g/mL ± 0.003 and a refractive index of 1.490 ± 0.009.

### 2.2. Chemical Composition

The chemical composition shows more presence of α- phellandrene at 43.16%, followed by trans-sesquisabinene hydrate with 8.23% and elemol with 7.21%. A total of 28 compounds were identified, out of which 27 were detected in the oil, resulting in 98.96%, and 1.04% was unidentified. 70% of the compounds correspond to terpene hydrocarbons, 27% to oxygenated sesquiterpenes and 2% to hydrocarbon sesquiterpenes. These data differ from research previously conducted in essential oil in Peru [8]. The complete chemical composition of the oil can be seen in Table 1.

### 2.3. Assessment of the Antimicrobial Activity

The results of the antimicrobial activity performed using the microdilution method shows variable activity in essential oil, which is considered as absent when it appears in concentrations higher than 20.000 µg/mL; it is low when the activity is between 20.000 and 5.000 µg/mL; it is strong or moderate when the activity occurs between 5.000 and 500 µg/mL; and it is very strong when the activity is less than 500 µg/mL [18].

Table 2 presents the results obtained in the 12 strains used in the research. There are remarkable results in which the activity is considered very strong, i.e., with values of minimum inhibitory concentration below 500 µg/mL; in this research, these values are observed in the following bacteria and yeasts: *Staphylococcus aureus* ATCC 6328, *Streptococcus mutans* ATCC 25175, *Candida albicans* ATCC 10231 and *Candida tropicalis* ATCC 13803.

### 2.4. Antimicrobial Bioautography

**Figure 1:**
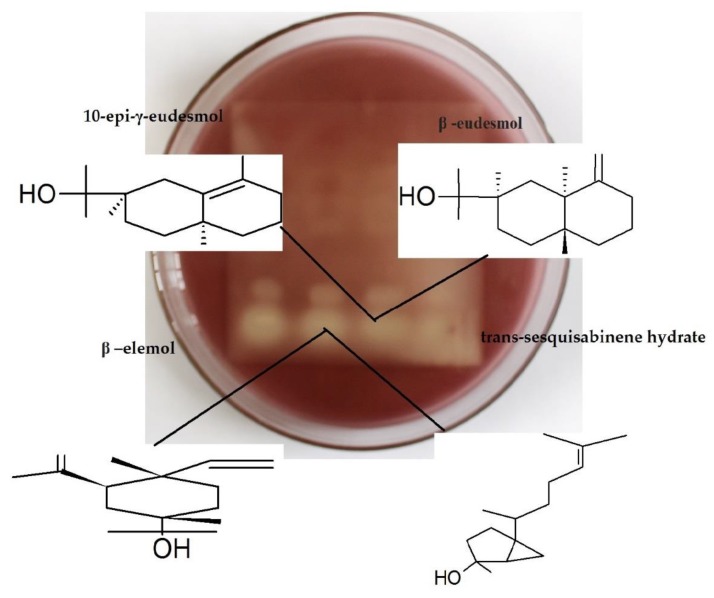
Antimicrobial bioautography against *Staphylococcus aureus*, Gram-positive bacteria.

**Figure 2:**
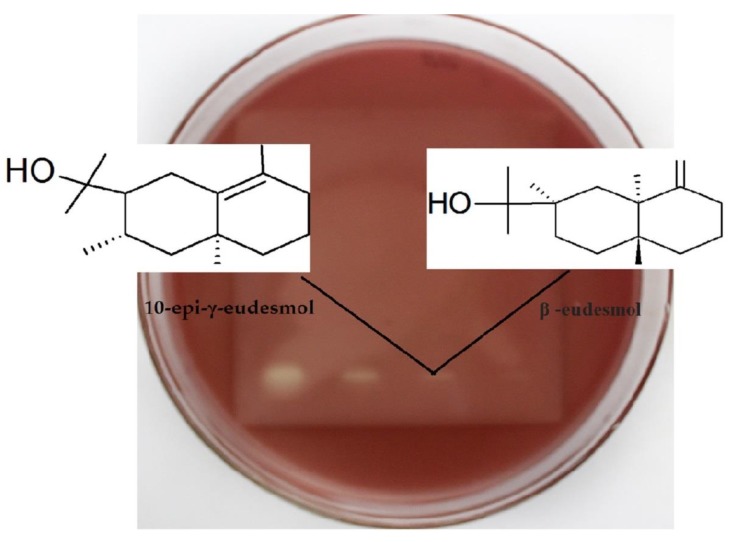
Antimicrobial bioautography against *Escherichia coli*, Gram-negative bacteria.

Bioautography shows intense antimicrobial activity at retention factors (Rf) of 0.02 and 0.04, with intense yellow coloration. However, the evaluation in the chromatographic plate containing the Gram-negative bacteria and the visualization of the molecules responsible for the activity become clearer and delimited in the molecules located at a retention factor of 0.04. In Figure 1 and Figure 2 can be observed the regions where the activity occurred for Gram-positive bacteria (*S. aureus)* and Gram-negative bacteria (*Escherichia coli*).

The four molecules involved correspond to the group of hydroxylated sesquiterpenes (molecules with 15 carbon atoms and OH group). In region Rf 0.02 are located: elemol (7.21%) and trans-sesquisabinene hydrate (8.23%). In region Rf 0.04 are found: β–eudesmol (3.49%) and 10-epi-γ-eudesmol (1.07%). HP-TLC plates show increased activity in Rf 0.04.

There is very little information in the scientific literature regarding this type of molecules and their antimicrobial potential; it should be considered that especially the most active molecules such as eudesmol and 10-epi-γ-eudesmol are not very abundant in the oil, and they have a very high antimicrobial activities.

## 3. Discussion

*P. barbatum* is used by the Andean people of Ecuador as for its disinfectant qualities, especially to control infections of the female genitalia. The first noteworthy result is that of the high concentration of essential oil in silver, close to 1% in fresh material, i.e., it is a species-rich in aromatic active ingredients, which could be a good indication for a holding sustained resource for industrial purposes.

The results of the minimum inhibitory concentration conducted by the microdilution method show different results for the bacteria and yeasts used, most have activity between 500-5.000 µg/mL, which implies that your activity is between strong or moderate. Ultimately, essential oil is antibacterial for a wide spectrum of microorganisms. In this study, results whose minimum inhibitory concentration value were less than 500 µg/mL are highlighted, such as: *S. aureus* 264 µg/mL, *S. mutans* 132 µg/mL; and the two yeasts *C. albicans* 132 µg/mL and *C. tropicalis* 264 µg/mL. The two yeasts are common in infections of the female genitalia [19], which would confirm the ancestral use of the species.

The most abundant component in the essential oil is α- phellandrene with 43.16%, which is not described as a molecule with antimicrobial potential; however, the high percentage of oxygenated sesquiterpenes close to 27% makes it interesting. There are few studies of biological activity related with these compounds and they are not used as reference antimicrobial molecules, unlike some oxygenated monoterpenes such as terpineol [20,21], thymol [22,23] or 1, 8 cineol [24], which are known by these qualities. This study showed different results in the chemical composition in comparison with the research has in Peru, were the most abundant compounds were: 2-methylene-4,5 dioxy-propiophenone and methoxy-4,5-methylenedioxy-propiophenone isomer II [8], in that study it was not evaluated the retention index. Antimicrobial bioautography shows the highest oil activity in those retention factors (Rf) rich in hydroxylated sesquiterpenes. In TLC performed with Gram-positive bacteria, the activity is very high, not allowing a differentiation of molecules; however, in the TLC containing the Gram-negative bacteria, the least intense activity reveals the specific position and relates to two molecules such as those responsible, β–eudesmol and 10-epi-γ-eudesmol. Subsequent evaluations with these compounds would be expected to confirm their activity, which could be interesting alternatives to the widespread use of antibiotics. The essential oil of the species could be considered as an active ingredient in intimate hygiene detergents and antimicrobial cosmetic products.

## 4. Material and Methods 

### 4.1. Plant Material

Leaves of *P. barbatum* were collected in the Armenian Metropolitan Park, in the city of Quito, in the following location: latitude: -0.283333, longitude: -78.4667, altitude 2537 m.a.s.l

### 4.2. Extraction of the Essential Oil

The method used was hydrodistillation, using a Clevenger device of 500 mL capacity. 100 gr of *P. barbatum* fresh leaves in good condition were used, the distillation time was 3 hours. The extractions were made for quintuplicate to obtain a promedium value. 

### 4.3. GC/MS and GC/FID Analyses

The composition of essential oils was analyzed by gas chromatography coupled to mass spectrometry, each individual molecule was quantified using GC/FID, triplicating each chromatographic test and calculating its relative area.

A chromatograph team brand Bruker model SCION 432 was used with a chromatographic column Zebron ZB-5MS (5% -phenyl-95% dimethyl) polysiloxane), with a length of 30 m, a thickness of 0.25 mm and a film thickness of 0.25 m, the column was directly coupled to a mass spectrometer brand Bruker model EVOQ. The haul gas was 99.9999% of pure helium at a flow of 1 mL/min, and split-ratio of 1:25.

The sample was prepared by diluting 10 µL of essential oil in 990 µL of dichloromethane. The analysis started at 45 °C, reaching 100 °C at a rate of 1 °C per minute, then it reached 250 °C at a speed of 5 °C per minute, staying at this temperature for 15 minutes for a total analysis time of 90 minutes.

Mass spectrometer conditions were: ionization energy: 70 eV; emission current: 10 µAmp; scan rate: 1 scan/s; mass range: 35-400 Da; trap temperature: 220 °C; Transfer line temperature: 260 °C.

A gas chromatograph Varian 3900 with an FID detector was used, using the Zebron ZB-5MS column. The operating conditions for gas chromatographs were reported above. FID temperature was 250 °C. The oil percentage composition was performed by the normalization method from the GC peak areas, without using correction factors and was the average of three injections [25].

### 4.4. Identification of Compounds 

The NIST 2001 Mass Spectra Database was used for the identification of molecules [26]. In addition, retention index rates (RI) of each compound were calculated by comparing a series of C_8_-C_30_ alkanes, and comparing the theoretical arithmetic retention rates, contrasting them with the scientific literature of the Adams database [17].

### 4.5. Antimicrobial Activity

The methodology used was microdilution, in which the bacteria or yeast is in contact with essential oil solutions in decreasing concentrations ranging from 5000 µg/ mL to 10 µg/ mL. In the same concentration ranges, two reference natural substances were used: *Thymus vulgaris* essential oil and citral, 2,3,5,-triphenyltetrazolium chloride (TTC) was used as an activity indicator. Positive controls were used: erythromycin 5 mg/mL for Gram-positive bacteria; gentamicin 10 mg/mL in Gram-negative bacteria and clotrimazole 6 mg/mL for yeasts. Each assay was triplicated. 

The bacteria used in this essay were: *E. coli* ATCC 25922, *Pseudomonas aeruginosa* ATCC 9027, *Proteus vulgaris* ATCC 6380, *Klebsiella oxytoca* ATCC 8724, Gram-positive bacteria; *S. aureus* ATCC 6328, *Enterococcus faecalis* ATCC 29212, *Listeria grayi* ATCC 19120, *Staphylococcus epidermidis* ATCC 14990, *Micrococcus luteus* ATCC 4698, *S. mutans* ATCC 25175, Gram-negative bacteria; and *C. albicans* ATCC 10231 and *C. tropicalis* ATCC 13803, yeasts.

### 4.6. Antimicrobial Bioautography

The separation of the compounds by high-resolution thin layer chromatography HP-TLC was performed using merk brand edify gel plates with fluorescence factor F 254. The solvent used was n-hexane, used as a solvent in the separation of sesquiterpenes [27]. The sample was prepared by dissolving 50 µL of essential oil in 950 µL of methanol. We added 10, 7.5, 2.5 µL of the solution on the plate with the help of an automatic Linomat IV planting device. The objective of volume reduction is to show activity when the concentration of compounds is lowered, as described in previous research [13].

The bacteria used in the trial were those whose results of antibacterial activity were the best: Gram-positive bacteria (*S. aureus*) and the Gram-negative bacteria *E. coli*, at a concentration of 1.5 × 10^8^ formers of colonies, and were diluted 50 times in the culture medium which contained a solution of (TTC) as an indicator of microbial prevalence.

The mixture of the medium (still liquid), bacteria and indicator was added until the chromatographic plate was covered, was kept at 0 °C for 2 hours, and then was incubated at 35 °C for 48 hours. Antibacterial activity was considered to exist in those Rf, which change the coloration from red to yellow.

Chemical components of each active region were analyzed, extracting them from the chromatographic plate use 2 mL of dichloromethane, ultrasound and centrifugation to extract the compounds of the solid phase. It was finally was analyzed by GC/MS, under the same analysis conditions as the essential oil.

### 4.7. Statistical Analysis.

Relative standard deviations and statistical significance (student’s test; *p* < 0.05), one way ANOVA and LSD post hoc Fisher´s honest significant difference test, were given, where appropriate, for all data collected. All computations were made using the statistical software STATISTICA 6.0.

## 5. Conclusions

The study proves that the ancestral knowledge of Quichua people is verified by demonstrating that the abundant essential oil that *P. barbatum* has is a biological resource with high antimicrobial potential. Hydroxylated sesquiterpenes and their antimicrobial potential could be considered as antimicrobial molecules from essential oils. In this case, these molecules require further evaluation but in an individual way and with a wider range of microorganisms.

## Figures and Tables

**Table 1:** Chemical composition of the essential oil of *Piper barbatum.*

Compound	Relative área (%)	RI lit ^a^	RI exp ^b^
α- thujene	0.65 ± 0.00	924	926
α- pinene	5.63 ± 0.05	932	931
camphene	0.33 ± 0.00	946	947
sabinene	2.49 ± 0.23	969	969
β – pinene	0.18 ± 0.00	974	976
myrcene	1.09 ± 0.00	988	991
α- phellandrene	43.16 ± 0.29	1002	1007
delta-3-carene	4.60 ± 0.04	1008	1012
limonene	7.04 ± 0.06	1024	1027
β-phellandrene	3.82 ± 0.17	1025	1029
p-mentha-2,4-diene	1.29 ± 0.04	1085	1087
bicyclogermacrene	0.73 ± 0.00	1500	1492
epi-cis-4-dihydrogafuran	0.64 ± 0.00	1499	1497
β –dihydro agarofuran	2.74 ± 0.00	1503	1508
β –curcumene	0.69 ± 0.00	1514	1513
cubebol	0.15 ± 0.00	1514	1515
p-sesquisabinene hydrate	0.48 ± 0.04	1542	1445
β –elemol	7.21 ± 0.21	1548	1550
trans-sesquisabinene hydrate	8.23 ± 0.21	1542	1550
(E)-nerolidol	0.69 ± 0.03	1561	1565
germacrene D-4- ol	0.16 ± 0.00	1574	1578
no identificado	1.04 ± 0.00	-	-
10-epi-γ-eudesmol	1.07 ± 0.03	1622	1627
epi-1-cubenol	0.20 ± 0.00	1627	1630
eremoligenol	0.21 ± 0.00	1629	1632
β -eudesmol	3.49 ± 0.11	1649	1654
β-epi-bisabolol	0.26 ± 0.00	1670	1677
α-(-) bisabolol	1.73 ± 0.02	1685	1688

Note: ^a^ Literature retention index by Adams [17], ^b^ Experimental retention index comparing a series of C_8_-C_30_ alkanes. The percentages of each area had a standard derivation < 5.0 %.

**Table 2:** Values of the minimum inhibitory concentration of *P. barbatum* essential oil in the different microorganisms.

Microorganism	*P. barbatum* Essential Oil MIC Values in (μg/mL)	*T. vulgaris* Essential Oil MIC Values in (μg/mL)	Citral MIC Values in (μg/mL)	Positive Control^a^ MIC Values in (μg/mL)
**Gram-negative bacteria**				
***Escherichia coli*** **ATCC 25922**	528	245	464	50
***Pseudomonas aeruginosa*** **ATCC 9027**	528	122	464	50
***Proteus vulgaris*** **ATCC 6380**	2110	122	464	25
***Klebsiella oxytoca*** **ATCC 8724**	2110	245	9283	25
**Gram-positive bacteria**				
***Staphylococcus aureus*** **ATCC 6328**	264	245	232	25
***Enterococcus faecalis*** **ATCC 29212**	528	122	464	50
***Listeria grayi*** **ATCC 19120**	2110	122	464	50
***Staphylococcus epidermidis*** **ATCC 14990**	528	500	232	25
***Micrococcus luteus*** **ATCC 4698**	528	245	464	50
***Streptococcus mutans*** **ATCC 25175**	132	61	232	25
**Yeasts**				
***Candida albicans*** **ATCC 10231**	132	61	116	40
***Candida tropicalis*** **ATCC 13803**	264	122	116	40

Note: ^a^ erythromycin for Gram-positive bacteria; gentamicin for Gram-negative bacteria and clotrimazole for yeasts.
